# Precision in Brief: The Bayesian Hurst–Kolmogorov Method for the Assessment of Long-Range Temporal Correlations in Short Behavioral Time Series

**DOI:** 10.3390/e27050500

**Published:** 2025-05-06

**Authors:** Madhur Mangalam, Aaron D. Likens

**Affiliations:** Division of Biomechanics and Research Development, Department of Biomechanics, and Center for Research in Human Movement Variability, University of Nebraska at Omaha, Omaha, NE 68182, USA; alikens@unomaha.edu

**Keywords:** detrended fluctuation analysis, fractal fluctuation, fractional, Hurst–Kolmogorov process, long-range correlation, physiology, variability

## Abstract

Various fields within biological and psychological inquiry recognize the significance of exploring long-range temporal correlations to study phenomena. However, these fields face challenges during this transition, primarily stemming from the impracticality of acquiring the considerably longer time series demanded by canonical methods. The Bayesian Hurst–Kolmogorov (HK) method estimates the Hurst exponents of time series—quantifying the strength of long-range temporal correlations or “fractality”—more accurately than the canonical detrended fluctuation analysis (DFA), especially when the time series is short. Therefore, the systematic application of the HK method has been encouraged to assess the strength of long-range temporal correlations in empirical time series in behavioral sciences. However, the Bayesian foundation of the HK method fuels reservations about its performance when artifacts corrupt time series. Here, we compare the HK method’s and DFA’s performance in estimating the Hurst exponents of synthetic long-range correlated time series in the presence of additive white Gaussian noise, fractional Gaussian noise, short-range correlations, and various periodic and non-periodic trends. These artifacts can affect the accuracy and variability of the Hurst exponent and, therefore, the interpretation and generalizability of behavioral research findings. We show that the HK method outperforms DFA in most contexts—while both processes break down for anti-persistent time series, the HK method continues to provide reasonably accurate *H* values for persistent time series as short as N=64 samples. Not only can the HK method detect long-range temporal correlations accurately, show minimal dispersion around the central tendency, and not be affected by the time series length, but it is also more immune to artifacts than DFA. This information becomes particularly valuable in favor of choosing the HK method over DFA, especially when acquiring a longer time series proves challenging due to methodological constraints, such as in studies involving psychological phenomena that rely on self-reports. Moreover, it holds significance when the researcher foreknows that the empirical time series may be susceptible to contamination from these processes.

## 1. Introduction

The fractal Hurst exponent, *H*—named by Mandelbrot in honor of Edwin Hurst’s pioneering work in hydrology [1]—is a robust measure quantifying the strength of long-range temporal correlations in time series [2]. Specifically, *H* quantifies how the variations in measurements change across progressively longer timescales, showing how the correlation among sequential measurements might change over longer timescales. The Hurst exponent is a fractal-scaling estimate of power-law decay in autocorrelation, represented by

(1) $$\begin{array}{c} \rho_{k}=\frac{{\left|k+1\right|}^{2H}-{2|k|}^{2H}+{\left|k-1\right|}^{2H}}{2}≃\frac{H(2H-1)}{k^{2-2H}},\;k=0,\;1,\;\ldots . \end{array}$$

The Hurst exponent indicates the degree of persistence (typically between 0.5 and 1.0) or anti-persistence (typically between 0 and 0.5) in the time series. The Hurst exponent has gained popularity in various fields, such as meteorology [3,4,5], economics [6,7,8,9,10], ethology [11,12], bioinformatics [13,14,15], and physiology [16,17,18,19]. In the behavioral sciences, it has been effectively employed in making inferences in various topics, like postural control [20,21,22], coordination [23,24], cognition [25,26,27,28,29], and perception–action [30,31,32]. It has also been used to distinguish between healthy and pathological cardiovascular functioning [33,34,35] and in the examination of movement systems [36,37,38,39,40,41]. Furthermore, it is becoming a standard statistic in creating and assessing rehabilitative interventions [42,43,44].

While Equation (1) describes the autocorrelation function of fractional Gaussian noise (fGn)—a widely accepted model for fractal time series with long-range temporal correlations—it is important to emphasize that fGn is not the only type of process with this property. $1/f^{\beta }$ noise, often referred to as 1/f noise or pink noise, is among the most commonly used models for fractal time series exhibiting power-law correlations. These processes also possess a well-defined Hurst exponent *H* and display asymptotic power-law decay in their autocorrelation functions [45]. Computational simplicity is a key reason for their popularity: generating 1/f noise is often more straightforward than generating fGn, making it a practical surrogate in the modeling and simulation of complex biological or psychological time series. By acknowledging this broader class of fractal processes, we highlight that the Hurst exponent and long-range dependence extend well beyond the specific case of fGn.

Biological and psychological work acknowledges the importance of investigating long-range temporal correlations to study phenomena [46,47,48,49,50,51,52,53]. Although recent studies have demonstrated the fractal properties of several psychological or behavioral variables, such as mood [54], self-esteem [55], and self-control [56], when examined over time, the transition towards exploring these correlations encounters challenges, primarily due to the impracticality of obtaining the significantly longer time series required by canonical methods. The divide between researchers’ theoretical aspirations and the practical reality of experimentation underscores the need for a method capable of accurately estimating the fractal exponent using short time series. This challenge becomes even more critical in the behavioral sciences, given that measurements of behavioral processes are often much noisier than strictly physical quantities. Bridging this gap necessitates a methodology to navigate these complexities and reasonably estimate the fractal exponent.

The most widely used method of estimating *H* is detrended fluctuation analysis (DFA) [57,58]. DFA has been widely used due to its ability to detect long-range temporal correlations in non-stationary time series and prevent the false detection of long-range temporal correlations [59,60]. However, it has several limitations, such as not accurately assessing the strength of long-range temporal correlations when the time series is short [61,62,63,64], producing a positive bias in its central tendency, and large dispersion [65,66,67,68,69,70]. DFA also requires at least 500 measurements to estimate *H*, which limits its applicability when a longer time series is not practical or feasible [67]. Additionally, DFA is sensitive to the time series length, which can result in overestimating *H*, mainly when used with short time series [66,71].

A less common method of estimating *H* is the Bayesian approach, which builds from the Hurst–Kolmogorov (HK) process, initially developed for hydrology [72]. With this method, Tyralis and [72] proposed a Bayesian-inspired technique that defines the posterior distribution from which the *H* value is sampled. The ways in which the HK and DFA methods perform calculations are quite different, with the HK method based on Bayes’ theorem and DFA determining the Hurst exponent directly from the time series data. In a previous study, we compared the performance of the two methods using simulated and real-world time series [73]. We found that the HK method outperformed the DFA in several ways, including its ability to detect long-range temporal correlations accurately, minimal dispersion around the central tendency, and a point estimate unaffected by the length of the measurement time series or its underlying Hurst exponent. We also found that the HK method balanced the risks of type I and type II errors in downstream statistical testing, making it a better choice overall for experimental work. Therefore, we encouraged the systematic application of the HK method to assess the strength of long-range temporal correlations in empirical time series in the behavioral sciences.

However, detecting and quantifying long-range temporal correlations in empirical time series is often challenging due to the presence of “artifacts”. The most common types of contaminants in empirical data that corrupt the long-range temporal correlations include the following.

- **Additive white Gaussian noise (awGn).** awGn is the most common contaminant in physiological records. Sometimes, awGn might occur due to inaccuracies in the measurement equipment—for instance, when recording electromyography [74,75] or an electrocardiogram in an intensive care unit [76]. At other times, awGn can be inherent in the measured system itself—for instance, the cardiac system [77,78], in which case the presence of awGn can be informative about the system’s condition, e.g., to detect arterial fibrillation [78,79].
- **Fractional Gaussian noise (fGn).** Although a less common contaminant than awGn, fGn often corrupts physiological signals. Sources of this contaminant often include similar systems; for instance, speech recordings of one person often become corrupted by fGn from surrounding speakers or echo from the same person’s speech [80,81].
- **Short-range correlations.** Temperature records constitute the most prominent and intuitive examples of measurements contaminated with short-range correlations characterized by strong persistence at the timescale of a few (usually <10) days superimposed on the long-range temporal correlations inherent in variability in weather conditions [82,83,84]. In the behavioral sciences, fractal fluctuations rarely appear in isolation in empirical time series and could be contaminated with various short-range correlated processes [85,86,87].
- **Trends.** Again, temperature records provide a convenient example of measurements contaminated with trends [88]. Due to experimental constraints, fatigue, etc., trends also ubiquitously contaminate behavioral measurements [89]. For instance, the stride length gradually increases or decreases along with long-range temporal correlations when a person starts or stops, respectively, walking on a treadmill, and the reaction time might increase due to increasing cognitive fatigue.

These artifacts can affect the accuracy and variability of the Hurst exponent and, therefore, the interpretation and generalizability of behavioral research findings. As a result, multiple methods have been developed to adapt the DFA algorithm to handle these issues more effectively when present in empirical data [90,91,92,93,94,95,96]. While we know that the HK method outperforms DFA when time series are uncontaminated [73], how these contaminants affect the estimation of *H* when using the HK method is still unclear.

In this paper, we study the performance of the HK and DFA methods in estimating the Hurst exponents of synthetic long-range correlated time series in the presence of additive white Gaussian noise, fractional Gaussian noise, short-range correlations, and various periodic and non-periodic trends.

## 2. Theoretical Background

### 2.1. Estimating the Hurst Exponent Using the HK Method

As noted above, a recently introduced Bayesian approach to estimating *H* [72] shows remarkable promise in addressing fundamental limitations with DFA. Previous work demonstrated that the HK method outperforms DFA in several contexts [73]. Below, we provide a brief overview of the HK method, while referring the reader to the foundational work for additional mathematical details and proofs [72]. Our notation generally follows the original work.

The foundation for the method originates in the definition of fGn as an instance of a process with Hurst–Kolmogorov (HK) properties [97] defined as

(2) $$\begin{array}{c} \rho_{k}=\frac{{\left|k+1\right|}^{2H}-{2|k|}^{2H}+{\left|k-1\right|}^{2H}}{2}≃\frac{H(2H-1)}{k^{2-2H}},\;k=0,\;1,\;\ldots , \end{array}$$

where *H* is the Hurst exponent, *k* is the time lag, and $\rho_{k}$ is the autocorrelation. This asymptotic expression reveals that the sign of the autocorrelation is determined by the term H(2H−1): $\rho_{k}$ is negative for H<0.5, zero for H=0.5, and positive for H>0.5. That is, when H=0.5, $\rho_{k}=0$ for all k>0, corresponding to uncorrelated white noise; when 0<H<0.5, the series is anti-persistent with short-term reversals; and, when 0.5<H<1, the series is positively correlated, with $\rho_{k}$ decaying slowly as *k* increases.

The HK method is a Bayesian approach to estimating *H* [72]. In the foundational work, Tyralis and Koutsoyiannis [72] derived a method to sample from the posterior distribution of *H* given by

(3) $$\begin{array}{c} \pi \left(\varphi |x_{n}\right)\propto {\left|R_{n}\right|}^{-1/2}{\left[e_{n}^{T}R_{n}^{-1}e_{n}x_{n}^{T}R_{n}^{-1}x_{n}-{\left(e_{n}^{T}R_{n}^{-1}e_{n}\right)}^{2}\right]}^{-(n-1)/2}{\left(e_{n}^{T}R_{n}^{-1}e_{n}\right)}^{n/2-1}. \end{array}$$

The natural logarithm of Equation (3) is then given by

(4) $$\begin{array}{c} \ln\pi (\varphi |x_{n})\propto \frac{1}{2}\ln|R_{n}|-\frac{n-1}{2}\ln\left[e_{n}^{T}R_{n}^{-1}e_{n}x_{n}^{T}R_{n}^{-1}x_{n}-{\left(e_{n}^{T}R_{n}^{-1}e_{n}\right)}^{2}\right]+\frac{n-2}{2}\ln\left(e_{n}^{T}R_{n}^{-1}e_{n}\right), \end{array}$$

where $R_{n}$ is the autocorrelation matrix with elements $r_{i,j}$, where i,j=1, 2, 3, …, n; $e_{n}\;=\;{\left(1,\;1,\;1,\;\ldots ,\;1\right)}^{T}$ is a vector of ones with *n* elements; |…| denotes a determinant; the superscript of −1 in $R_{n}^{-1}$ is a matrix inverse; and the superscript *T* is a matrix transpose. For a given $x_{t}$ and $\rho_{k}$, the quadratic forms of the symmetric, positive definite autocorrelation matrix are used to derive the matrix products on the right-hand side of Equation (4) (Levinson Algorithm; Algorithm 4.7.2, Golub and Van Loan [98], p. 235).

Accept–reject algorithms are standard tools for sampling from posterior distributions and serve as the backbone when implementing the HK method [99]. Let f(x) be a probability density function (PDF) from which it is difficult to sample. f(x) is the “target distribution” and can be sampled using Monte Carlo methods. First, we sample a simpler “proposal distribution” Mg(x) that has the same domain as f(x), and *M* is a constant that is large enough to ensure that g(x)≥f(x). Theoretically, the proposal distribution, g(x), can be any number of distributions, such as uniform, truncated Gaussian, exponential, etc. However, the algorithm gains computational efficiency when the overall shape of g(x) is similar to *f*. Second, f(x) is evaluated at the value obtained by sampling g(x), the proposal distribution. Third, a sample is drawn from U(x)∼Uniform(0,Mg(x)). If U(x)≤f(x), then the value proposed by sampling g(x) is accepted as a valid sample. Otherwise, the proposal is rejected, and the algorithm is re-initialized. This process repeats until *n* samples are obtained, where *n* is the number of samples desired from the posterior distribution.

In the simulations reported in the following section, we employ this accept–reject algorithm to sample *H* from its posterior distribution (Algorithm A.5, Robert et al. [99], p. 49). The target distribution f(x) is Equation (4) and g(x)∼Uniform(0,1). The uniform distribution makes sense for g(x) because it shares the same domain of *H* and hence Equation (4), namely (0,1) [72]. A numerical optimization routine determines *M* by finding the maximum of Equation (4) as a function of *H*. The point estimate of *H* is then taken as the median of the posterior distribution of *H* (see Appendix A).

### 2.2. Estimating the Hurst Exponent Using DFA

DFA computes the Hurst exponent, *H*, quantifying the strength of long-range temporal correlations in series [57,58] using the first-order integration of *T*-length time series x(t):

(5) $$y\left(i\right)=\sum_{k=1}^{i}\left(x\left(k\right)-\bar{x(t)}\right),\;i=1,\;2,\;3,\;\ldots ,\;T.$$

DFA computes the root mean square (RMS, i.e., averaging the residuals) for each *k*th-order trend $y_{n,k}\left(t\right)$ fit to $N_{n}$ non-overlapping *n*-length bins to build a fluctuation function:

(6) $$f\left(v,n\right)=\sqrt{\frac{1}{N_{n}}\sum_{v=1}^{N_{n}}\left(\frac{1}{n}\sum_{i=1}^{n}{\left(y\left((v-1)n+i\right)-y_{v,k}\left(i\right)\right)}^{2}\right)},\;n=\left\{4,\;8,\;12,\;\ldots \right\}<\frac{T}{2}.$$

f(n) is a power law,

(7) $$f\left(n\right)∼n^{H},$$

where *H* is the scaling exponent estimable using logarithmic transformation:

(8) logf(n)=Hlogn.

We used the Davies–Harte algorithm [100] to generate fractional Gaussian noise (fGn), which can be tuned to exhibit varying degrees and directions of autocorrelation consistent with Equation (2). We generated fGn time series in the R [101] programming environment using the function fGn_sim() as part of the package “fractalRegression” [102]. The function fGn_sim() has two inputs: the time series length, *N*, and the Hurst exponent, *H*. We generated 1000 synthetic fGn time series for each combination of six different time series lengths (N=32, 64, 128, 256, 512, 1024) and nine different a priori known values of *H* (H=0.1, 0.2, …, 0.9). We submitted the time series to the HK methods in the *R* [101] programming environment using the inferH() function as part of the package “HKprocess” [103]. The function inferH() has two inputs: the time series, $x_{N}$, and the simulated sample size from the posterior distribution of *H*, *n*. We used *n* of 100; previous simulations have shown that n=100 provides an excellent trade-off between accuracy and computational efficiency, and n>>50 provides no further accuracy-related benefits [104]. We submitted the time series to the DFA in the *R* [101] programming environment using the function dfa() as part of the package “fractalRegression” [102]. A bin size range of [4,N/2] was used for the DFA in the present study, which is standard practice when using DFA [105,106,107,108,109].

Although a common implementation of DFA uses a fitting range between s=4 and s=N/2, there is increasing recognition that this choice may not always capture the true asymptotic behavior of long-range correlations. As discussed in Ref. [110], narrower intervals such as s∈[8,N/10] may provide more reliable estimates of *H* by avoiding small scales where the power-law regime may not yet be established. Indeed, as Ref. [111] emphasizes, the scaling behavior that DFA seeks to detect is asymptotic, and reliable estimates of *H* require the exclusion of small window sizes that may be affected by non-scaling dynamics, nonstationarities, or filtering artifacts. We acknowledge this important consideration and note that the sensitivity of *H* estimation to the choice of fitting range remains an open methodological challenge in analyzing finite, noisy time series. A systematic investigation of the fitting range sensitivity would help to determine robust intervals across applications and data types in future studies.

### 2.3. The Effects of Additive White Gaussian Noise

We examine the performance of the HK method and DFA in assessing the Hurst exponent for long-range correlated time series contaminated with additive white Gaussian noise (awGn), such that

(9) $$\begin{array}{c} y_{N}=x_{N}+Au_{N}, \end{array}$$

where $x_{N}$ is a long-range correlated time series, $u_{N}$ is an awGn time series characterized by zero mean and unit variance, and *A* defines the amplitude of the awGn time series added to $x_{N}$ and is allowed to vary from 0 to 1 with an increment of 0.1 (i.e., A=0, 0.1, 0.2, …, 1).

**Figure 1:**
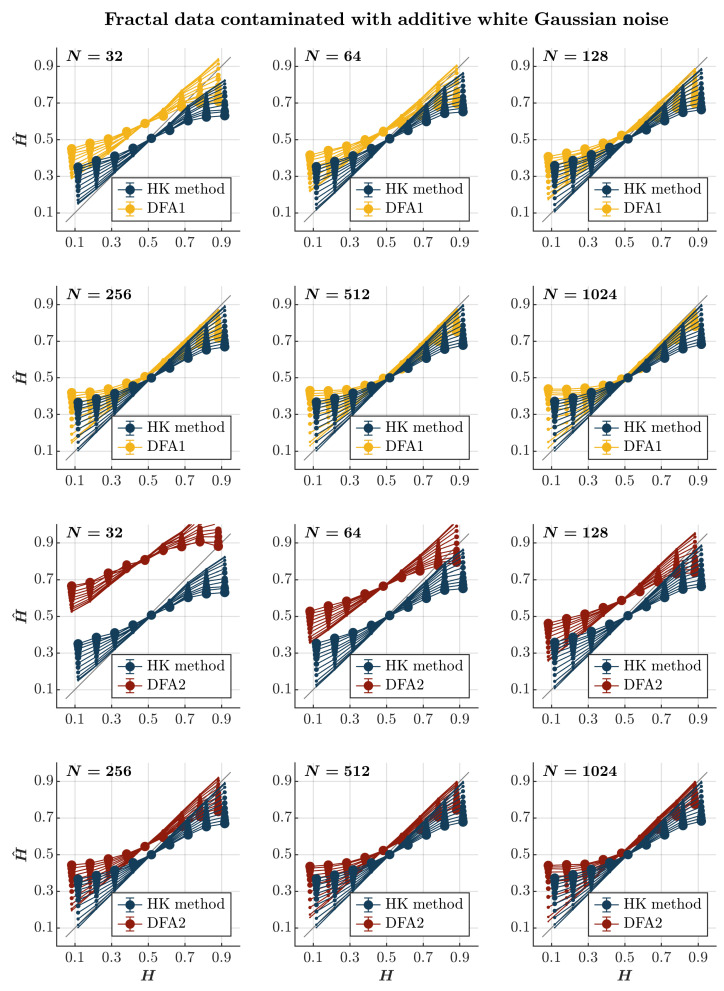
*Fractal data contaminated with awGn.* Each panel plots the mean estimated $\hat{H}$ using the HK method (*blue circles*) and DFA1 (*yellow circles*) and DFA2 (*red circles*) for 1000 synthetic time series of length N=32, 64, 128, 256, 512, 1024 with a priori known values of *H* to which Gaussian white noise of unit standard deviation was added with different weights ranging from 0 to 1 with an increment of 0.1. The size of the solid circles indicates the weight of the contaminant. The gray line indicates the ideal case when $\hat{H}=H$. Error bars indicate 95% CI.

Both the HK method and the first-order DFA overestimate $\hat{H}$ for anti-persistent time series (i.e., H<0.5) contaminated with awGn and underestimate *H* for persistent time series contaminated with awGn (0.5<H≤1; *blue and yellow circles*, respectively, in Figure 1). These effects are accentuated with the magnitude of the added awGn. Adding awGn to an anti-persistent time series will render it less anti-persistent, and adding awGn to a persistent time series will render it less persistent, which is quite intuitive. Nonetheless, the HK method yields more accurate $\hat{H}$ values for anti-persistent time series contaminated with awGn. In contrast, the first-order DFA yields more accurate $\hat{H}$ values for persistent time series highly contaminated with awGn. This contrast highlights the typical tendency of the first-order DFA to overestimate long-range temporal correlations [66,71], which does not seem to be the case with the HK method, as noted previously [73]. Notably, these effects do not seem to depend on the time series length, reflecting the length-independent effect of added awGn on long-range temporal correlations in synthetic time series. The second-order DFA consistently overestimates $\hat{H}$ in short time series, regardless of their persistence characteristics. However, it produces a more accurate estimation of $\hat{H}$ for persistent time series, particularly when they are both sufficiently long and heavily contaminated with awGn (*red circles* in Figure 1).

Note that awGn is a particular case of fGn with H=0.5. So, in principle, $\hat{H}$ can be roughly anticipated to be an average of the “pure” *H* corresponding to the long-range correlated time series $x_{N}$ and the awGn time series $u_{N}$ with H=0.5, with this latter value weighted with the employed amplitude *A*, i.e., $\hat{H}\propto \left(1-A\right)H+A\times 0.5$. This is roughly what is observed in most of the results in Figure 1, where increasing contamination by awGn gradually shifts the estimated $\hat{H}$ values closer to 0.5, consistent with the attenuated influence of the original long-range correlations.

### 2.4. The Effects of fGn

We examine the performance of the HK method and DFA in assessing the Hurst exponent for long-range correlated time series contaminated with fGn, such that

(10) $$\begin{array}{c} y_{N}=x_{N}+Ap_{N}, \end{array}$$

where $x_{N}$ is a long-range correlated time series, $p_{N}$ is an fGn time series with H=0.9 and characterized by zero mean and unit variance, and *A* defines the amplitude of the fGn added to $x_{N}$ and is allowed to vary from 0 to 1 with an increment of 0.1 (i.e., A=0, 0.1, 0.2, …, 1).

**Figure 2:**
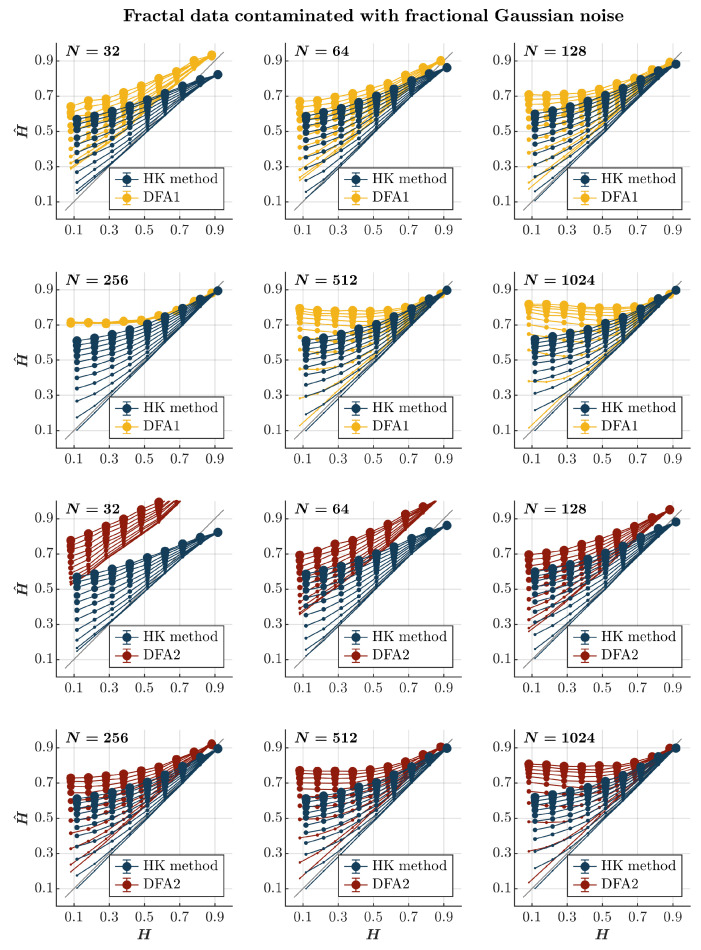
*Fractal data contaminated with fGn.* Each panel plots the mean estimated $\hat{H}$ using the HK method (*blue circles*) and DFA1 (*yellow circles*) and DFA2 (*red circles*) for 1000 synthetic time series of length N=32, 64, 128, 256, 512, 1024 with *a priori* known values of *H* to which fGn of unit standard deviation was added with different weights ranging from 0 to 1 with an increment of 0.1. The size of the solid circles indicates the weight of the contaminant. The gray line indicates the ideal case when $\hat{H}=H$. Error bars indicate 95% CI.

Both the HK method and the first-order DFA overestimate $\hat{H}$ for both anti-persistent and persistent time series contaminated with fGn, independently of the time series length (*blue and yellow circles*, respectively, in Figure 2), confirming the theoretical expectation that adding fGn—which is long-range correlated—will increase the strength of the long-range temporal correlations in the original time series. Likewise, the extent of overestimation is accentuated with the magnitude of the added fGn. The overestimation of *H* is particularly problematic for anti-persistent time series, for which both methods yield $\hat{H}$ values indicating persistence. Although of no practical use in this situation, the HK method is marginally better than the first-order DFA in providing substantially larger but comparatively less biased estimates of $\hat{H}$, highlighting the typical tendency of the first-order DFA to overestimate long-range temporal correlations [66,71]. Furthermore, the HK method yields a marginally overestimated yet more accurate $\hat{H}$ than the first-order DFA for highly persistent time series (H>0.7). The second-order DFA significantly amplifies the overestimation of $\hat{H}$ in extremely brief time series (N=32), irrespective of their persistence characteristics. Nevertheless, it results in a milder overestimation of $\hat{H}$ for anti-persistent time series with N≥64 and an underestimation of $\hat{H}$ for persistent time series, especially in cases where they are heavily contaminated with fGn (*red circles* in Figure 2).

### 2.5. The Effects of Short-Range Correlations

We examine the HK method and DFA’s performance in assessing the Hurst exponent for long-range correlated time series contaminated with short-range correlations. We created short-range correlations by filtering random data. We chose short-range correlations described by the first-order autoregressive (AR1) process

(11) $$\begin{array}{c} {\left(1-B\right)}^{d}s_{N}=a_{1}s_{N-1}+u_{N-1}, \end{array}$$

where *B* is the backshift operator and d=0.25 is the fractional differencing parameter for the generated AR1 process $s_{N}$, $a_{1}=0.1$ is the model parameter, and $u_{N}$ is an awGn time series characterized by zero mean and unit variance. The autocorrelation function decays with a time constant $\tau =-\frac{1}{\ln|a_{1}|}$. The result is

(12) $$\begin{array}{c} y_{N}=x_{N}+As_{N}, \end{array}$$

where $x_{N}$ is a long-range correlated time series, $s_{N}$ is the vector of short-range correlations, and *A* defines the amplitude of the short-range correlations added to $x_{N}$ and is allowed to vary from 0 to 1 with an increment of 0.1 (i.e., A=0, 0.1, 0.2, …, 1).

**Figure 3:**
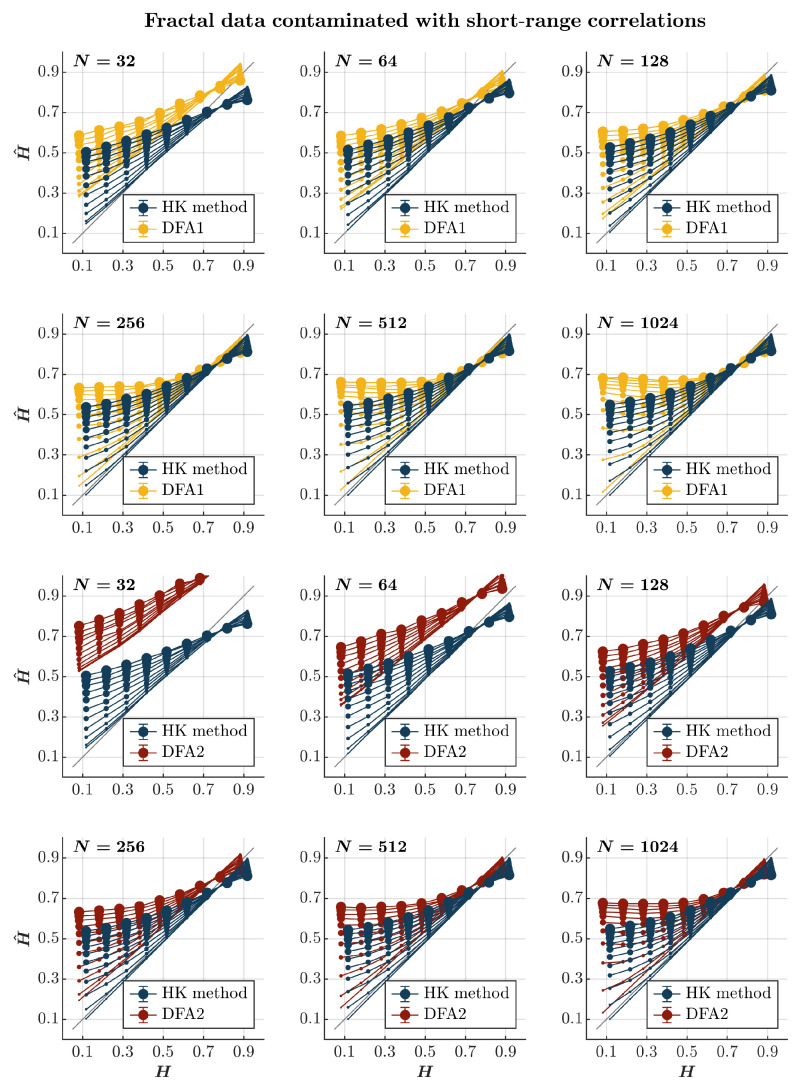
*Fractal data contaminated with short-range correlations.* Each panel plots the mean estimated $\hat{H}$ using the HK method (*blue circles*) and DFA1 (*yellow circles*) and DFA2 (*red circles*) for 1000 synthetic time series of length N=32, 64, 128, 256, 512, 1024 with a priori known values of *H* to which short-range correlations of unit standard deviation were added with different weights ranging from 0 to 1 with an increment of 0.1. The size of the solid circles indicates the weight of the contaminant. The gray line indicates the ideal case when $\hat{H}=H$. Error bars indicate 95% CI.

Contaminating time series with short-range correlations produces more nuanced effects on the estimation accuracy of the HK method and DFA. Both the HK method and the first-order DFA overestimate $\hat{H}$ for anti-persistent (H<0.5) and less persistent (0.5<H<0.7) time series contaminated with short-range correlations (*blue and yellow circles*, respectively, in Figure 3) confirming the theoretical expectation that adding short-range correlations will reduce the anti-persistence. The magnitude of the added short-range correlations accentuates the extent of this overestimation. The overestimation of $\hat{H}$ is particularly problematic for anti-persistent time series. Both methods yield $\hat{H}$ values indicating persistence, especially when the time series is heavily contaminated. Again, while of no practical use, the HK method is marginally better than DFA in providing substantially larger but comparatively less biased estimates of $\hat{H}$. In contrast, both methods underestimate $\hat{H}$ for highly persistent time series (H>0.7), reflecting the fact that adding short-range correlations to long-range correlated time series accentuates the contribution of short-range correlations in the estimation of the Hurst exponent. Finally, the two methods do not seem to differ when estimating $\hat{H}$ for highly persistent time series with N≥128 contaminated with short-range correlations. The second-order DFA significantly amplifies the overestimation of $\hat{H}$ except for the highly persistent time series with N≥512 heavily contaminated with short-range correlations (*red circles* in Figure 3). In these scenarios, the second-order DFA seems to provide a slightly more accurate estimation of $\hat{H}$ than the HK method.

### 2.6. The Effects of Cyclical Trends

We examine the performance of the HK method and DFA in assessing the Hurst exponent for long-range correlated time series contaminated with cyclical trends simulated by a harmonic function

(13) $$\begin{array}{c} y_{N}=x_{N}+A\sin\frac{2\pi N}{\tau }, \end{array}$$

where $x_{N}$ is a long-range correlated time series and *A* defines the amplitude of the cyclical trend added to $x_{N}$ and is allowed to vary from 0 to 1 with an increment of 0.1 (i.e., A=0, 0.1, 0.2, …, 1). We used τ=365 to simulate the short-term cyclical trend and τ=36,500 to simulate the long-term cyclical trend.

**Figure 4:**
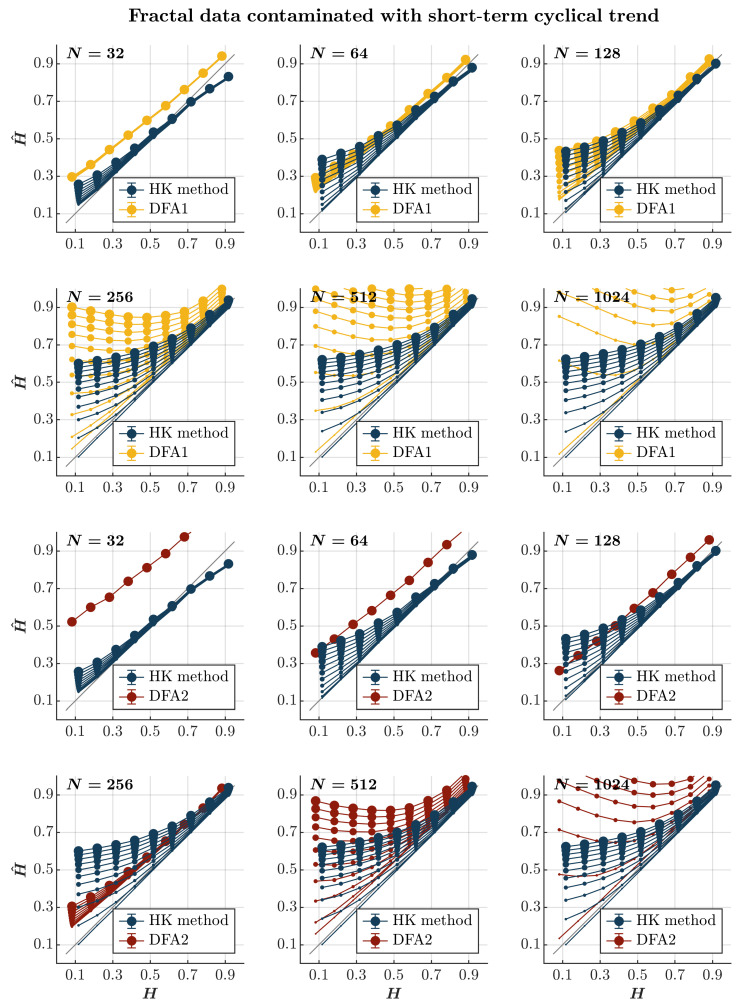
*Fractal data contaminated with a short-term cyclical trend.* Each panel plots the mean estimated $\hat{H}$ using the HK method (*blue circles*) and DFA1 (*yellow circles*) and DFA2 (*red circles*) for 1000 synthetic time series of length N=32, 64, 128, 256, 512, 1024 with a priori known values of *H* to which short-term cyclical trend $\sin\frac{2\pi N}{\tau }$ with τ=365 was added with different weights ranging from 0 to 1 with an increment of 0.1. The size of the solid circles indicates the weight of the contaminant. Note that the curves for different levels of contamination overlap in several instances. The gray line indicates the ideal case when $\hat{H}=H$. Error bars indicate 95% CI.

Consistent with previous observations [110], contaminating time series with a short-term cyclical trend—the term comparable to the timescale of the entire time series—leads to an overall overestimation of $\hat{H}$, independently of persistence or the lack thereof, using both the HK method and the first-order DFA (*blue and yellow circles*, respectively, in Figure 4). Notably, while this overestimation grows with the magnitude of the cyclical trend and the time series length for both methods, it renders the first-order DFA useless, yielding $\hat{H}$ values exceeding 1 for anti-persistent time series (H<0.5) of length N≥512—which is longer than the recommended length for DFA [112]. Although the HK method also deems anti-persistent time series persistent in the presence of a short-term cyclical trend, it estimates $\hat{H}$ for more persistent time series (H≥0.7) with acceptable accuracy. The second-order DFA markedly exacerbates the overestimation of $\hat{H}$ in time series with N≤128; intriguingly, the $\hat{H}$ estimated by the second-order DFA remains unaffected by the contamination amplitude. In contrast, for time series with N≥256, the second-order DFA yielded a less inflated $\hat{H}$ compared to the first-order DFA, yet it still performed inferiorly to the HK method (*red circles* in Figure 4).

**Figure 5:**
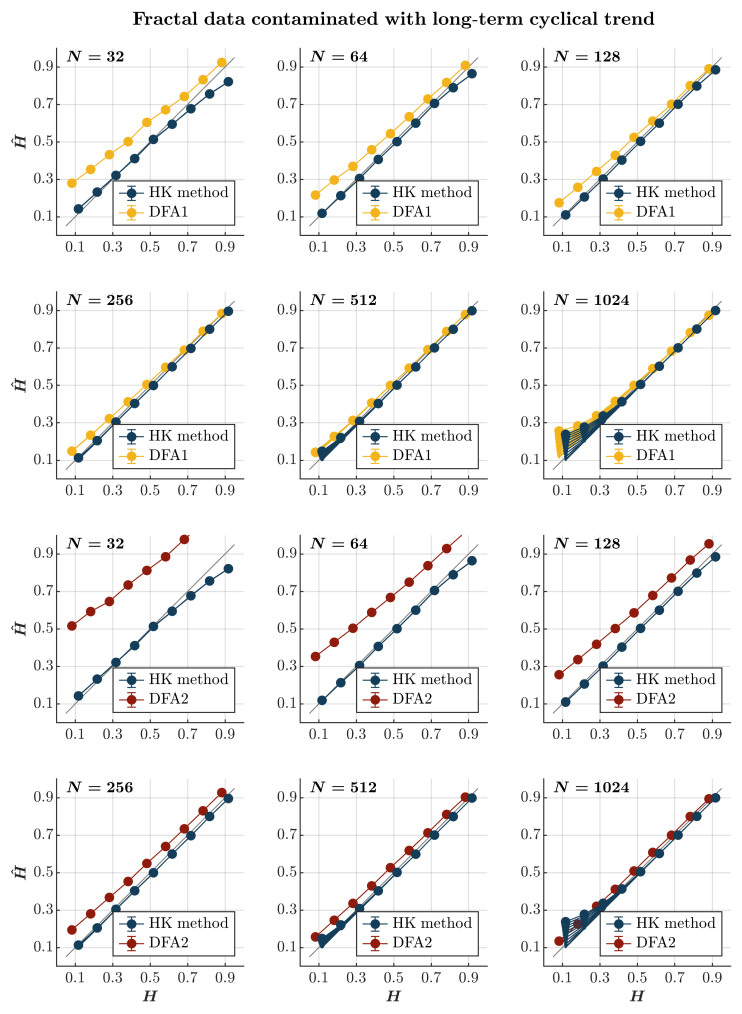
*Fractal data contaminated with a long-term cyclical trend.* Each panel plots the mean estimated $\hat{H}$ using the HK method (*blue circles*) and DFA1 (*yellow circles*) and DFA2 (*red circles*) for 1000 synthetic time series of length N=32, 64, 128, 256, 512, 1024 with a priori known values of *H* to which long-term periodic trend $\sin\frac{2\pi N}{\tau }$ with τ=36,500 was added with different weights ranging from 0 to 1 with an increment of 0.1. The size of the solid circles indicates the weight of the contaminant. Note that the curves for different levels of contamination overlap in several instances. The gray line indicates the ideal case when $\hat{H}=H$. Error bars indicate 95% CI.

Contaminating time series with a long-term cyclical trend—the term being orders of magnitude longer than the timescale of the entire time series—leads to the marginal overestimation of $\hat{H}$ for more anti-persistent time series (H≥0.7) of length N≥512 when using both the HK method and the first-order DFA (*blue and yellow circles*, respectively, in Figure 5). In this case, the HK method consistently outperforms the first-order DFA, estimating $\hat{H}$ increasingly more accurately with the time series length than the first-order DFA. The second-order DFA intensifies the overestimation of $\hat{H}$ in the time series, with the estimated $\hat{H}$ remaining impervious to the contamination amplitude, but performs less effectively than the HK method (*red circles* in Figure 5).

### 2.7. The Effects of Linear Trends

We examine the performance of the HK method and DFA for long-range correlated time series contaminated with a linear trend, such that

(14) $$\begin{array}{c} y_{n}=x_{n}\pm A\left(0.005\cdot n\right), \end{array}$$

where $x_{n}$ is a long-range correlated time series and *A* defines the amplitude of the linear trend 0.005·n added to or subtracted from $x_{n}$ and is allowed to vary from 0 to 1 with an increment of 0.1 (i.e., A=0, 0.1, 0.2,…, 1).

**Figure 6:**
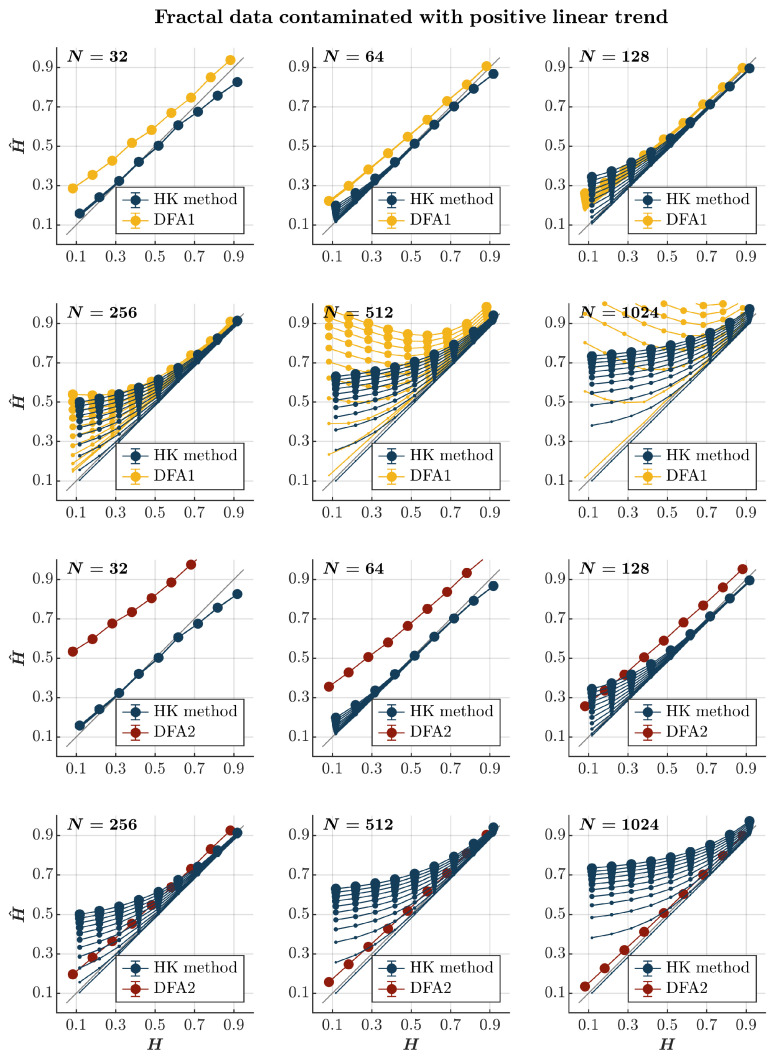
*Fractal data contaminated with a positive linear trend.* Each panel plots the mean estimated $\hat{H}$ using the HK method (*blue circles*) and DFA1 (*yellow circles*) and DFA2 (*red circles*) for 1000 synthetic time series of length N=32, 64, 128, 256, 512, 1024 with a priori known values of *H* to which positive linear trend 0.005i(i=1, 2, 3, …, N) was added with different weights ranging from 0 to 1 with an increment of 0.1. The size of the solid circles indicates the weight of the contaminant. Note that the curves for different levels of contamination overlap in several instances. The gray line indicates the ideal case when $\hat{H}=H$. Error bars indicate 95% CI.

**Figure 7:**
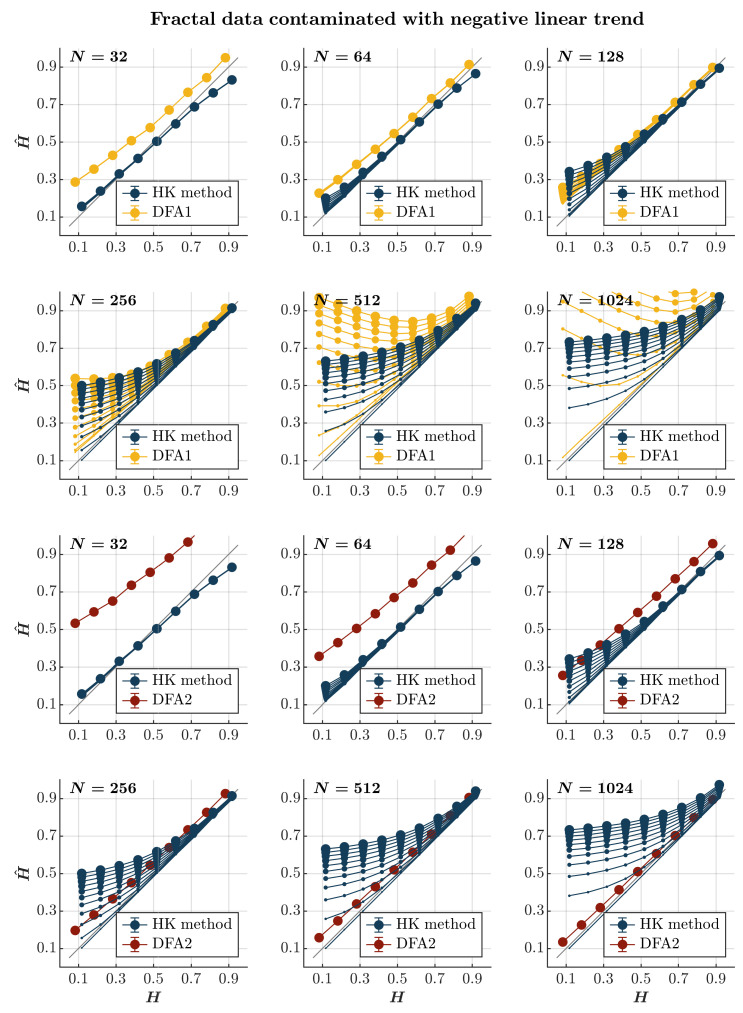
*Fractal data contaminated with a negative linear trend.* Each panel plots the mean estimated $\hat{H}$ using the HK method (*blue circles*) and DFA1 (*yellow circles*) and DFA2 (*red circles*) for 1000 synthetic time series of length N=32, 64, 128, 256, 512, 1024 with a priori known values of *H* to which negative linear trend −0.005i(i=1, 2, 3, …, N) was added with different weights ranging from 0 to 1 with an increment of 0.1. The size of the solid circles indicates the weight of the contaminant. Note that the curves for different levels of contamination overlap in several instances. The gray line indicates the ideal case when $\hat{H}=H$. Error bars indicate 95% CI.

Consistent with previous observations [110], contaminating time series with a positive or negative linear trend consistently results in the overall overestimation of $\hat{H}$ using both the HK method and the first-order DFA, regardless of the presence or absence of persistence (*blue and yellow circles*, respectively, in Figure 6 and Figure 7). Although the HK method accurately estimates $\hat{H}$ for time series with N≤64, the overestimation amplifies with the linear trend’s magnitude and the time series length for both methods. For the first-order DFA, this overestimation renders it impractical, yielding $\hat{H}$ values reaching 0.5 for anti-persistent time series with N≥256 and surpassing 1 for anti-persistent time series with N≥512, exceeding the recommended length for DFA [112]. Despite the HK method misclassifying anti-persistent time series as persistent, especially for those with N≥256, it demonstrates acceptable accuracy in estimating $\hat{H}$ for more persistent time series (H≥0.7). However, even the HK method exhibits a progressively more overestimated $\hat{H}$ for these time series with an increasing time series length. On the other hand, the second-order DFA consistently results in an overall overestimation of $\hat{H}$ for time series with N≤128. However, it outperforms the HK method in accurately estimating $\hat{H}$ for time series with N≥256, regardless of whether the time series is contaminated with a positive or negative trend (*red circles* in Figure 6 and Figure 7).

### 2.8. The Effects of Quadratic Trends

We examine the performance of the HK method and DFA for long-range correlated time series contaminated with a quadratic trend such that

(15) $$\begin{array}{c} y_{n}=x_{n}\pm A\left(0.000005\cdot n^{2}\right), \end{array}$$

where $x_{n}$ is a long-range correlated time series and *A* defines the amplitude of the quadratic trend $0.000005\cdot n^{2}$ added to or subtracted from $x_{n}$ and is allowed to vary from 0 to 1 with an increment of 0.1 (i.e., A=0, 0.1, 0.2, …, 1).

**Figure 8:**
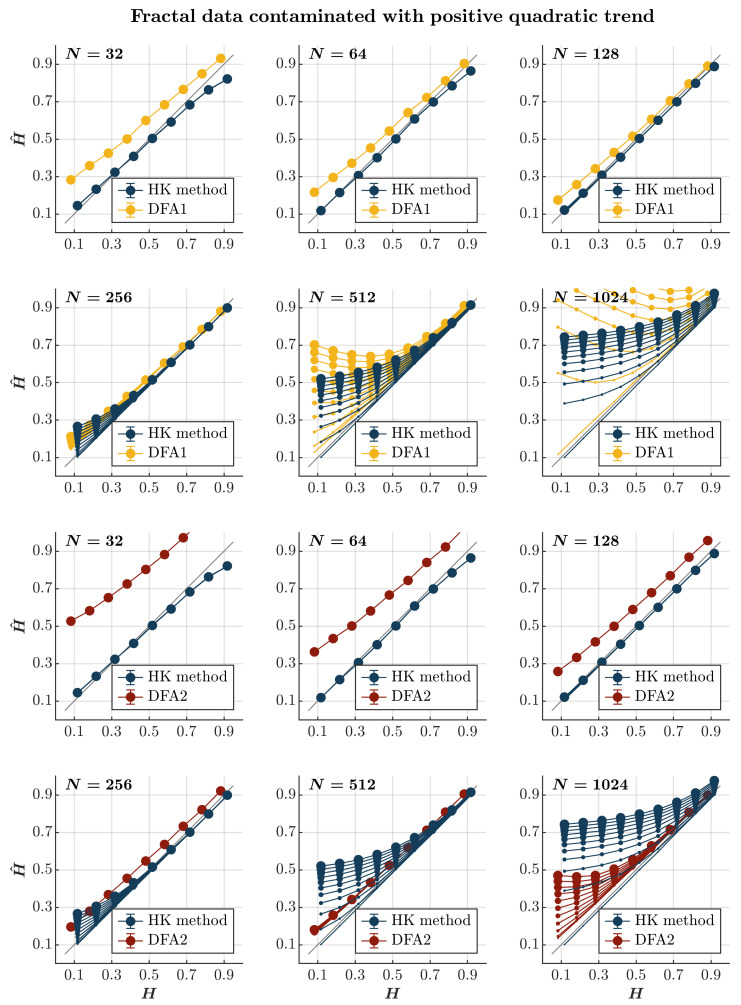
*Fractal data contaminated with a positive quadratic trend.* Each panel plots the mean estimated $\hat{H}$ using the HK method (*blue circles*) and DFA1 (*yellow circles*) and DFA2 (*red circles*) for 1000 synthetic time series of length N=32, 64, 128, 256, 512, 1024 with a priori known values of *H* to which positive quadratic trend $0.000005i^{2}\;\left(i=1,\;2,\;3,\;\ldots ,\;N\right)$ was added with different weights ranging from 0 to 1 with an increment of 0.1. The size of the solid circles indicates the weight of the contaminant. Note that the curves for different levels of contamination overlap in several instances. The gray line indicates the ideal case when $\hat{H}=H$. Error bars indicate 95% CI.

**Figure 9:**
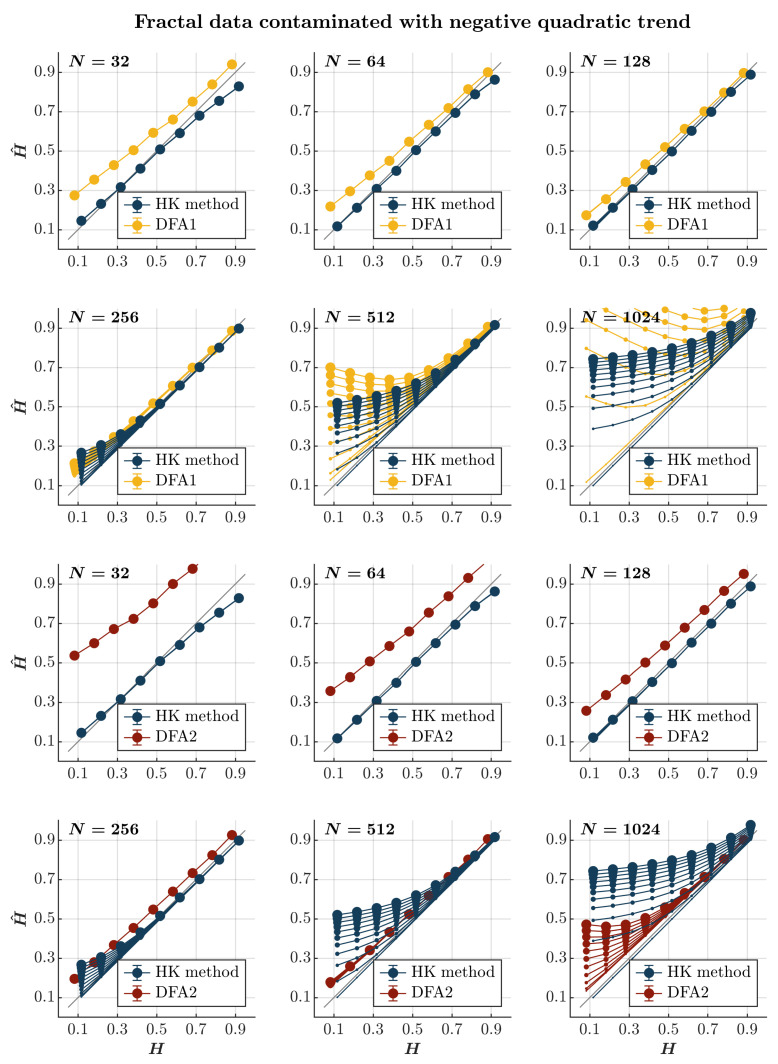
*Fractal data contaminated with a negative quadratic trend.* Each panel plots the mean estimated $\hat{H}$ using the HK method (*blue circles*) and DFA1 (*yellow circles*) and DFA2 (*red circles*) for 1000 synthetic time series of length N=32, 64, 128, 256, 512, 1024 with a priori known values of *H* to which negative quadratic trend $-0.000005i^{2}\;\left(i=1,\;2,\;3,\;\ldots ,\;N\right)$ was added with different weights ranging from 0 to 1 with an increment of 0.1. The size of the solid circles indicates the weight of the contaminant. Note that the curves for different levels of contamination overlap in several instances. The gray line indicates the ideal case when $\hat{H}=H$. Error bars indicate 95% CI.

Consistent with previous observations [110], contaminating time series with a positive or negative quadratic trend yields effects akin to those induced by a linear trend, albeit with a more gradual escalation dependent on the time series length. Specifically, introducing a positive or negative quadratic trend results in the pervasive overestimation of $\hat{H}$ when using both the HK method and the first-order DFA, regardless of persistence (*blue and yellow circles*, respectively, in Figure 8 and Figure 9). While this overestimation escalates with the magnitude of the quadratic trend and the time series length for both methods, the estimate remains reliable for a time series of length N∼256. Simultaneously, the HK method and the first-order DFA misclassify anti-persistent time series (H<0.5) with a length of N≥512 as persistent in a positive or negative quadratic trend. However, the HK method accurately estimates $\hat{H}$ for more persistent time series (H≥0.7), indicating superior overall performance when long-range correlated time series are contaminated with a positive or negative quadratic trend. On the contrary, the second-order DFA consistently results in an overall overestimation of $\hat{H}$ for time series with N≤128. Nevertheless, it surpasses the HK method in accurately estimating $\hat{H}$ for time series with N≥256, regardless of whether the contamination involves a positive or negative quadratic trend (*red circles* in Figure 8 and Figure 9).

### 2.9. When to Use the HK Process and First- and Second-Order DFA

**Figure 10:**
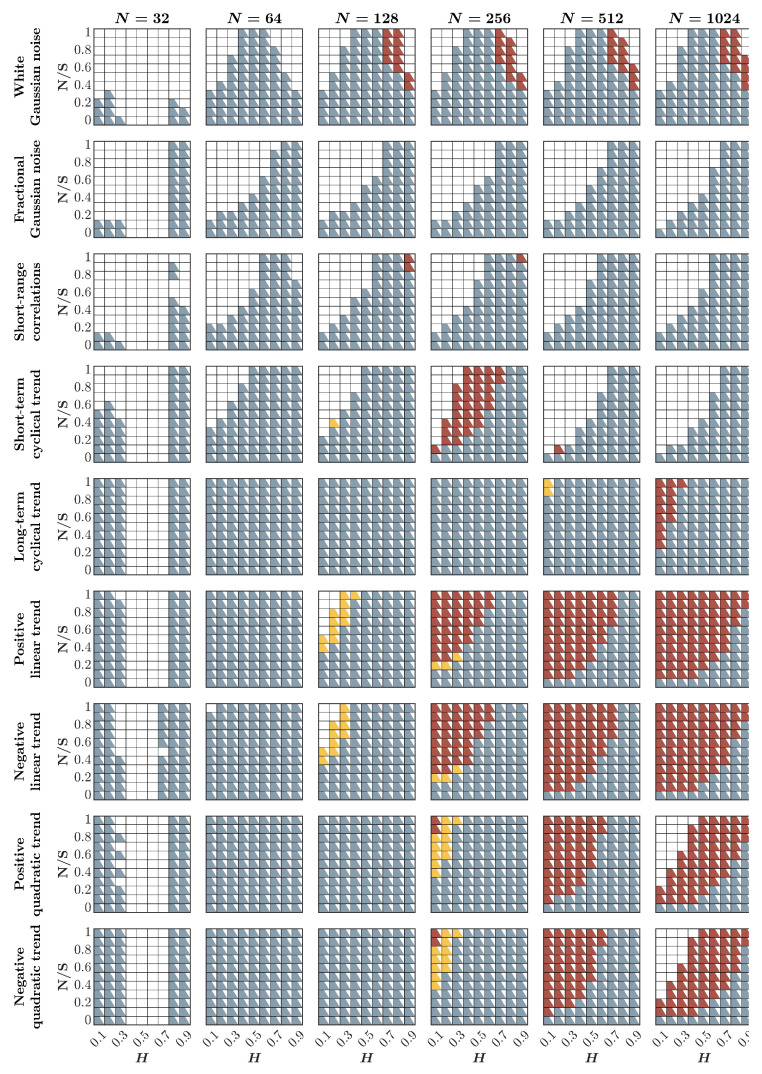
*Comparison of the Bayesian HK method, first-order DFA, and second-order DFA in estimating the fractal Hurst exponent, $\hat{H}$, in the presence of awGn, fGn, short-range-correlations, and linear and quadratic positive and negative trends.* Each panel indicates the method that estimates $\hat{H}$ most accurately based on the mean estimated $\hat{H}$ for 1000 synthetic time series of length N=32, 64, 128, 256, 512, 1024 with a priori known values of *H*, to which additive noise, short-range-correlations, or trends were added with different weights ranging from 0 to 1 with an increment of 0.1 (noise to signal ratio, $\frac{N}{S}$). *Blue, yellow, and red squares* indicate that the Bayesian HK method, DFA1, or DFA2, respectively, provides the most accurate estimation of $\hat{H}$. Only those cases for which the difference between the actual and estimated values, i.e., $\hat{H}∼H\leq 0.1$, are shown; white boxes indicate $\hat{H}∼H>0.1$, suggesting that none of these methods should be used to estimate $\hat{H}$ in these cases.

Figure 10 presents a comprehensive comparison of the Bayesian HK method, first-order DFA, and second-order DFA in the estimation of the fractal Hurst exponent, $\hat{H}$, under the influence of various contaminants, such as awGn, fGn, and short-range-correlations, as well as linear and quadratic positive and negative trends. When dealing with very short time series, i.e., N≤64, the HK process consistently demonstrates more accurate estimates of $\hat{H}$ than the first- and second-order DFA. This trend persists when the time series is contaminated with short-range correlations, regardless of the time series length. In cases involving other contaminants, it becomes evident that second-order DFA outperforms the HK method and first-order DFA in accurately estimating $\hat{H}$ when the time series is sufficiently long, i.e., N≥256, and is anti-persistent or weakly persistent with significant contamination. However, if the time series is either uncontaminated or inherently persistent and only mildly contaminated, the HK process unequivocally surpasses both first- and second-order DFA in performance for short and long time series alike, providing credence to our proposal of adopting the HK method over DFA in behavioral science [73].

Nevertheless, there are instances where none of these methods may accurately estimate $\hat{H}$ with sufficient precision—specifically, when $\hat{H}∼H\leq 0.1$. This is predominantly observed in scenarios with exceptionally brief time series (N≤64) or when the data are heavily contaminated with short-range correlations and short-term cyclical trends (depicted as *white boxes* in Figure 10). Under these circumstances, it is advisable to exercise caution and consider abstaining from using any of these methods.

## 3. Discussion

Biological and psychological work acknowledges the importance of scrutinizing long-range temporal correlations to study phenomena [46,47,48,49,50,51,52,53]. Despite recent demonstrations of the fractal properties in psychological and behavioral variables like mood [54], self-esteem [55], and self-control [56], explorations of these correlations encounter challenges over time. A comprehensive understanding of the temporal dynamics in mood disorders bears immense potential for the refinement of assessment methodologies and diagnostic criteria and the development of prevention and treatment strategies. Unfortunately, analyzing such data for long-range temporal correlations has remained challenging. These challenges result in a large gap between researchers’ theoretical ambitions and the pragmatic challenges of experimentation, highlighting the necessity of a method that aids in precisely estimating the fractal exponent with short time series. This imperative is particularly pronounced in the behavioral sciences, where the data are often affected by various noise sources. Specifically, the difficulty in analyzing this data type is its frequent unsuitability for traditional DFA. The data that we come across are usually of low quality, short, and basic. Making this situation even more challenging is the fact that the methods typically used to study short time series are known for their accuracy in the long run but struggle with issues like measurement noise or changes in the data caused by their short length compared to the characteristic timescales of the processes involved, as well as potential interference from external signals. We demonstrate that the HK method effectively overcomes these limitations by providing accurate estimates of the Hurst exponent even for short time series containing as few as 64 samples. Our findings reveal remarkable alignment between the Hurst exponent obtained through the HK method and the known Hurst exponent of synthetic time series with a length as short as 64 samples. This consistency holds despite contaminants such as awGn, fGn, short-range correlations, and various periodic and non-periodic trends, particularly in cases where the time series exhibits inherent long-range temporal correlations. In stark contrast, the first-order DFA consistently yields inaccurate Hurst exponents for both short time series and those with substantial actual Hurst exponents. However, it is noteworthy that the second-order DFA outperforms the HK method under specific conditions—namely, when the time series is sufficiently long (N≥256), characterized by anti-persistence or weak persistence with significant contamination. Consequently, users must judiciously assess the extent of contamination in the time series before selecting either the HK method or DFA for Hurst exponent estimation.

The estimation of the Hurst exponent from empirical data introduces various challenges that impact accuracy and dispersion—factors such as trends [110,113,114], nonstationarity [113,115], nonlinearity [116], and instances where the Hurst exponent exceeds one [59,111,117,118] contribute to this complexity. Consequently, multiple attempts have been made to enhance the adaptability of the DFA algorithm for empirical data exhibiting one or more of these challenges [90,91,92,93,94,95,96]. Our study bypasses this need and these developments by demonstrating that the HK method proves robust against these contaminants, delivering a satisfactory estimation of the Hurst exponent even for time series as short as 64 values. Future investigations could delve into assessing the sensitivity of the HK method to strong trends, nonstationarity, nonlinearity, and instances of larger-than-one *H* individually or in combination. Replicating these findings across simulated and empirical datasets will be essential, paving the way for a more comprehensive pipeline to objectively determine whether the HK process or DFA is the preferred choice for Hurst exponent estimation.

In essence, our study brings renewed optimism for those performing complexity analysis. The inability to effectively identify potential long-range temporal correlations in short data sequences has constrained a significant portion of behavioral research, relying on qualitative assessments or self-reports, despite recurring observations of these phenomena exhibiting self-similar fractal patterns. The outcomes presented herein robustly affirm the effectiveness of the Bayesian HK method in discerning long-range temporal correlations, even in extremely short time series, amid the presence of contaminants and trends arising from unwanted or contextual factors. However, it is crucial to note that the real litmus test for the Bayesian HK method lies in its application to real-life self-report data. This critical avenue for future research holds the promise of further cementing the credibility of the Bayesian HK process, thus propelling its integration into behavioral sciences.

## Data Availability

No data were used for the research described in this article.

## Appendix A. Code Snippet for the Estimation of the Bayesian Hurst Exponent Using the HK Method in R

Listing A1 provides the R snippet for the estimation of the Bayesian Hurst exponent of simulated time series of various lengths with various Hurst values.

**Listing A1.** R code snippet for estimation of the Bayesian Hurst exponent of a time series.
